# Supplementary material for: In-Hospital Mortality and Morbidity in Cancer Patients with COVID-19: A Nationwide Analysis from the United States
Source: Cancers (Basel). 2022 Dec 30;15(1):222. doi: 10.3390/cancers15010222 (PMC9818639; doi:10.3390/cancers15010222)
Supplement: Supplementary file 1 [file cancers-15-00222-s001.zip › cancers-2108450-supplementary.pdf]

Supplementary Appendix

In-Hospital Mortality and Morbidity in Cancer Patients with COVID-19: a Nationwide Analysis from the United States

Ziad Abuhelwa MD[1], Anas Alsughayer MD[1], Ahmad Y Abuhelwa PhD[2,3], Azizullah Beran MD[4], Wasef Sayeh MD[1], Waleed Khokher MD[1], Omar Sajdeya MD[1], Sadik Khuder DDS, MPH, PhD [1, 5,6], Ragheb Assaly MD[1,7]

1. Department of Medicine, University of Toledo, Toledo, OH 43606, USA
2. Department of Pharmacy Practice and Pharmacotherapeutics, College of Pharmacy, University of Sharjah, P.O. Box 27272, Sharjah, United Arab Emirates
3. Sharjah Institute for Medical Research, University of Sharjah, P.O. Box 27272, Sharjah, United Arab Emirates
4. Division of Gastroenterology and Hepatology, Indiana University, Indianapolis, IN 46202, USA
5. School of Population Health, University of Toledo, Toledo, OH 43614, USA
6. Department of Mathematics & Statistics, College of Natural Sciences and Mathematics, University of Toledo, Toledo, OH 43614, USA
7. Division of Pulmonology and Critical Care Medicine, Department of Medicine, University of Toledo, Toledo, OH 43606, USA

Corresponding author: Ziad Abuhelwa, MD | Department of Medicine, University of Toledo, Toledo, OH 43606, USA | Tel: +1-567-420-1600 | Email: ziad.abuhelwa@utoledo.edu | ORCID 0000-0002-0031-7150

Supplementary Table S1: ICD-10-CM codes for diseases and procedures used in the study

**Diagnosis/procedure**	**ICD-10-CM code**
COVID-19	U071
Lung cancer	C34, C39.9
Breast cancer	C50
Colorectal cancer	C18, C19, C20
Prostate cancer	C61
Leukemia	C91, C92, C93, C94, C95
Lymphoma	C81, C82, C83, C84, C85, C86, C88
Multiple myeloma	C90
Congestive heart failure	I110, I130, I50
Coronary artery disease	I25
Chronic pulmonary disease	J43, J44, J45, J455, J459, J47, J841, J848
Diabetes mellitus	E08, E09, E10, E11, E13
Hypertension	I10, I11X, I12X, I13XX, I15X, I16X,
Renal failure	N18, N19
Liver disease	K70, K71, K72, K73, K74, K75, K76, K77
Obesity	E66
Smoking	F172, Z720, Z87891
Alcohol abuse	F10
Drug abuse	F11, F12, F13, F14, F15, F16
Septic shock	R6521
Acute respiratory failure	J9600, J9601, J9602, J9620, J9621, J9622
Acute respiratory distress syndrome	J80
Mechanical ventilation	5A1935Z, 5A1945Z, 5A1955Z

Supplementary Table S2: Outcomes according to cancer type

	Mortality % (95%CI), *P*-value	Morbidity % (95%CI), *P*-value				Resource utilization mean (95%CI), *P*-value	
	**In-hospital mortality**	**Septic shock**	**ARF**	**ARDS**	**MV**	**LOS (days)**	**Total hospital charges ($)**
Lung (n=4,400)	20.91 (18.32-23.76), <0.001*	3.52 (2.48-4.98), 0.772	61.59 (58.25-64.83), 0.001*	3.75 (2.69-5.21), 0.045*	9.89 (8.1-12.01), 0.601	7.69 (7.17-8.21), 0.425	72,097 (65,731-78,463), 0.046*
Breast (n=3,340)	10.33 (8.23-12.9), 0.49	2.54 (1.59-4.04), 0.246	51.0 (46.84-54.35), 0.003*	4.19 (2.91-6.0), 0.226	7.49 (5.73-9.72), 0.088	7.46 (6.86-8.07), 0.959	72,803 (63,644-81,961), 0.211
Colorectal (n=1,675)	19.1 (15.21-23.71), <0.001*	3.88 (2.26-6.57), 0.586	55.82 (50.3-61.2), 0.913	4.18 (2.5-6.9), 0.386	12.24 (9.06-16.33), 0.082	8.47 (7.55-9.39), 0.034	83,099 (70,625-95,572), 0.47
Prostate (n=4,080)	19.0 (16.39-21.91), <0.001*	3.19 (2.18-4.64), 0.799	56.74 (53.34-60.08), 0.715	4.66 (3.39-6.37), 0.473	8.21 (6.48-10.36), 0.258	7.62 (7.11-8.14), 0.585	76,990 (68,374-85,606), 0.709
Leukemia (n=6,230)	16.45 (14.48-18.64), <0.001*	3.61 (2.65-4.9), 0.623	60.11 (57.26-62.9), 0.005*	5.86 (4.64-7.37), 0.321	10.19 (8.52-12.14), 0.36	8.32 (7.86-8.78), <0.001*	86,599 (77,179-96,020), 0.091
Lymphoma (n=4,320)	17.96 (15.55-20.66), <0.001*	5.67 (4.29-7.45), <0.001*	55.56 (52.21-58.85), 0.735	5.9 (4.5-7.71), 0.367	11.81 (9.8-14.15), 0.014*	8.47 (7.85-9.1), 0.002*	88,038 (78,021-98,055), 0.062
Myeloma (n=3,000)	18.33 (15.36-21.73), <0.001*	4.83 (3.4-6.84), 0.039*	54.17 (50.25-58.04), 0.321	5.5 (3.89-7.73), 0.765	11.83 (9.5-14.64), 0.037*	8.14 (7.52-8.75), 0.035*	94,002 (79,074-108,930), 0.033*

Abbreviation: CI; confidence interval, ARF; acute respiratory failure, ARDS; acute respiratory syndrome, MV; mechanical ventilation, LOS; length of hospital stay
* Statistical significance (*P*-vale <0.005)
